# Bis(2,2′-bipyrimidine-κ^2^ *N* ^1^,*N* ^1′^)palladium(II) bis­(tetra­fluoro­borate) acetonitrile monosolvate

**DOI:** 10.1107/S1600536812041591

**Published:** 2012-10-13

**Authors:** Adam Duong, James D. Wuest, Thierry Maris

**Affiliations:** a Département de Chimie, Université de Montréal, 2900 Boulevard Édouard-Montpetit, Montréal, Québec, Canada H3C 3J7

## Abstract

The reaction of [Pd(MeCN)_4_](BF_4_)_2_ with 2,2′-bipyrimidine (bpm) in MeCN–CHCl_3_ afforded the title compound, [Pd(C_8_H_6_N_4_)_2_](BF_4_)_2_·C_2_H_3_N. The asymmetric unit contains two half complexes, with the Pd^II^ atoms both lying on a twofold axis. Each metal atom adopts a tetra­hedrally distorted square-planar geometry. In the crystal, [Pd(bpm)_2_] dications are linked by C—H⋯N hydrogen bonds, forming chains parallel to the *b* axis. The chains are further linked by C—H⋯F and C—H⋯N inter­actions involving the tetra­fluoro­borate anions and acetonitrile mol­ecules. In this way, each chain interacts with six surrounding chains to generate the observed three-dimensional structure.

## Related literature

Similar Pd(bpm)_2_ complexes are unknown, but the subject of related dicationic adducts of Pd^II^ with 2,2′-bipyridyl (bpy) has been reviewed by Constable (1989 ▶) and McKenzie (1971 ▶), and the structures of representative analogues have been reported by Chieh (1972 ▶), Duong *et al.* (2011 ▶), Gao *et al.* (2010 ▶), Geremia *et al.* (1992 ▶), Hinamoto *et al.* (1972 ▶), Maeda *et al.* (1986 ▶), Milani *et al.* (1997 ▶), Stoccoro *et al.* (2002 ▶), Wehman *et al.* (1994 ▶) and Yue *et al.* (2008 ▶).
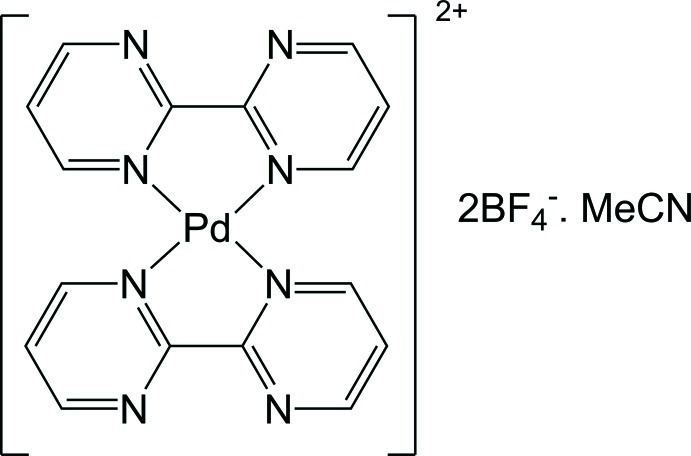


## Experimental

### 

#### Crystal data

[Pd(C_8_H_6_N_4_)_2_](BF_4_)_2_·C_2_H_3_N
*M* *_r_* = 637.41
Monoclinic, 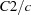

*a* = 18.0686 (4) Å
*b* = 18.1126 (4) Å
*c* = 14.8351 (3) Å
β = 108.613 (1)°
*V* = 4601.13 (17) Å^3^
*Z* = 8
Cu *K*α radiation
μ = 7.38 mm^−1^
*T* = 200 K
0.20 × 0.11 × 0.10 mm

#### Data collection

Bruker SMART 6000 diffractometer
Absorption correction: multi-scan (*SADABS*; Sheldrick, 2004 ▶) *T*_min_ = 0.311, *T*_max_ = 0.478
31076 measured reflections
4246 independent reflections
4006 reflections with *I* > 2σ(*I*)
*R*_int_ = 0.031

#### Refinement

*R*[*F* ^2^ > 2σ(*F* ^2^)] = 0.030
*wR*(*F* ^2^) = 0.081
*S* = 1.03
4246 reflections
347 parameters
H-atom parameters constrained
Δρ_max_ = 1.07 e Å^−3^
Δρ_min_ = −0.75 e Å^−3^

### 

Data collection: *SMART* (Bruker, 2003 ▶); cell refinement: *SAINT* (Bruker, 2003 ▶); data reduction: *SAINT*; program(s) used to solve structure: *SHELXS97* (Sheldrick, 2008 ▶); program(s) used to refine structure: *SHELXL97* (Sheldrick, 2008 ▶); molecular graphics: *SHELXTL* (Sheldrick, 2008 ▶) and *Materials Studio* (Accelrys, 2002 ▶); software used to prepare material for publication: *UdMX* (Maris, 2004 ▶) and *publCIF* (Westrip, 2010 ▶).

## Supplementary Material

Crystal structure: contains datablock(s) I, global. DOI: 10.1107/S1600536812041591/rz5009sup1.cif

Structure factors: contains datablock(s) I. DOI: 10.1107/S1600536812041591/rz5009Isup2.hkl

Supplementary material file. DOI: 10.1107/S1600536812041591/rz5009Isup3.cdx

Additional supplementary materials:  crystallographic information; 3D view; checkCIF report

## Figures and Tables

**Table 1:** Hydrogen-bond geometry (Å, °)

*D*—H⋯*A*	*D*—H	H⋯*A*	*D*⋯*A*	*D*—H⋯*A*
C1—H1⋯N11^i^	0.95	2.62	3.460 (4)	148
C6—H6⋯N15^ii^	0.95	2.45	3.237 (4)	140
C14—H14⋯N16^iii^	0.95	2.40	3.228 (4)	146
C2—H2⋯F7^iii^	0.95	2.38	3.282 (3)	159
C3—H3⋯F2^iv^	0.95	2.44	3.240 (3)	142
C8—H8⋯F3^v^	0.95	2.45	3.270 (4)	145
C13—H13⋯F6^vi^	0.95	2.37	2.982 (3)	122
C15—H15⋯F8^iv^	0.95	2.52	3.408 (4)	155
C18—H18*B*⋯F5^vii^	0.98	2.55	3.373 (5)	142

Symmetry codes: (i) 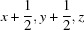; (ii) 
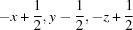
; (iii) 
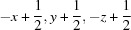
; (iv) 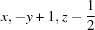; (v) 
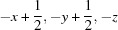
; (vi) 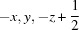; (vii) 
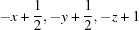
.

**Fig. 1.**
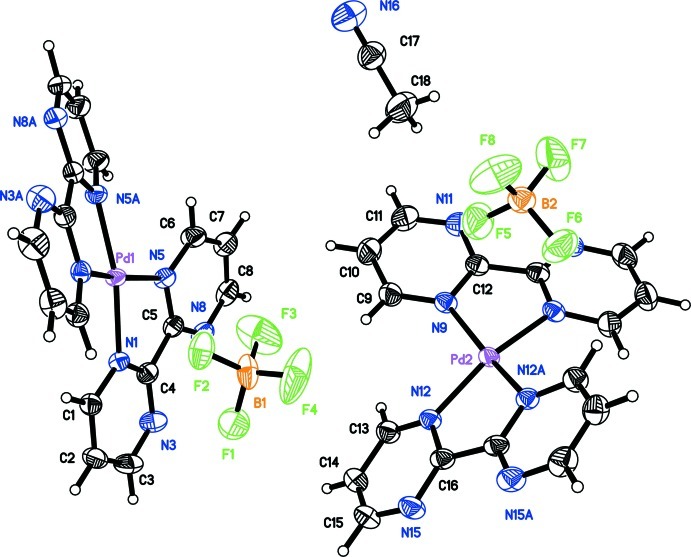
Molecular structure of the title compound, with atomic labels and 50% probability displacement ellipsoids for non-hydrogen atoms. Hydrogen atoms are drawn as a sphere of arbitrary radius. Unlabelled atoms in the cationic complexes are related by the symmetry operations 1 - x, y, -z + 1/2 for Pd1 and -x, y, -z + 1/2 for Pd2.

**Fig. 2.**
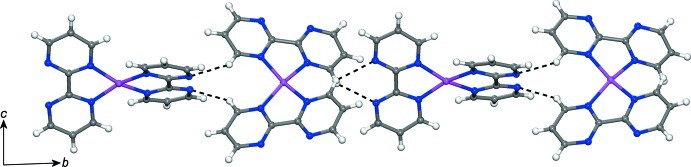
View along the a-axis of a chain of cationic complexes, with C—H···N interactions shown as broken lines.

**Fig. 3.**
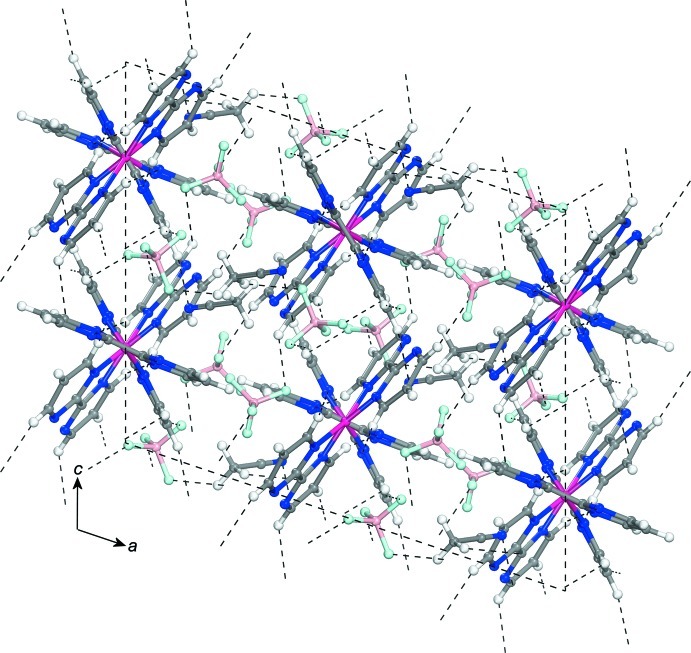
View along the b-axis of the unit-cell content. C—H···N and C—H···F contacts are shown as dashed lines.

## supplementary crystallographic information

### Comment

The title compound was obtained and characterized in the course of a study of complexes of Pd(II) with chelating heterocyclic ligands (Duong *et al.*, 2011). In the crystal structure, each Pd^II^ center is coordinated by two 2,2'-bipyrimidine (bpm) ligands to form two different distorted square-planar complexes (Figure 1). Distortions in such complexes arise because a normal square-planar geometry, which is inherently preferred by *d*^8^ metals, is opposed by steric interactions of the two ligands. As a result, related complexes of 2,2'-bipyridine (bpy) typically adopt one of two characteristic conformations: a so-called *twisted* geometry (involving a tetrahedral distortion of the metal center) and a *bow-step* deformation of the ligands themselves, as described by Constable (1989) and Milani *et al.* (1997). In the title compound, each of the two observed complexes incorporates approximately planar bpm ligands (maximum r. m. s. deviation 0.089 Å), and the geometry of coordination is twisted, with angles α of 21.52 (16)° and 25.80 (18)° between the N—Pd—N planes of the ligands. These values of α are similar to those found in analogous dicationic Pd^II^(bpy)_2_ complexes with twisted geometries, as reported by Chieh (1972), Geremia *et al.* (1992), Hinamoto *et al.* (1972), Milani *et al.* (1997), Stoccoro *et al.* (2002), and Wehman *et al.* (1994). In addition, the average Pd—N distance is normal [2.030 (2) Å] (McKenzie, 1971).

The structure consists of chains of Pd^II^(bpm)_2_ dications linked along the *b*-axis by C—H···N hydrogen bonds involving uncoordinated atoms of nitrogen (mean C—H···N distance: 3.348 (4) Å; Table 1), as shown in Figure 2. Within each chain, adjacent dications are arranged in an approximately orthogonal way (the dihedral angle between the N_4_ coordination cores is 89.01 (6)°). The tetrafluoroborate counterions are located between the chains and bridge them *via* weak C—H···F contacts to create the three-dimensional packing. Included molecules of MeCN are located close to the Pd^II^ centers, fill remaining volume, and engage in additional C—H···N interactions (Figure 3).

### Experimental

Solid [Pd(MeCN)_4_](BF_4_)_2_ (71 mg, 0.16 mmol) was added at 25 °C to a stirred mixture of MeCN (2.5 ml) and CHCl_3_ (2.5 ml) containing 2,2'-bipyrimidine (50 mg, 0.32 mmol). The resulting mixture quickly turned yellow, and a yellow solid began to precipitate. After the suspension had been stirred for 2 h, volatiles were removed by evaporation under reduced pressure, and the residual yellow solid was washed with MeCN. Crystals of the title complex were obtained in 70% yield by allowing vapors of MeCN to diffuse slowly into a solution of the yellow solid in DMSO.

### Refinement

All H-atoms were placed in calculated positions (C—H 0.95–0.98 Å) and were included in the refinement in the riding model approximation, with U(H) set to 1.2Ueq(C) for hydrogen atoms bonded to *sp*^2^-hybridized carbon atoms and 1.5Ueq(C) for hydrogen atoms bonded to *sp*^3^-hybridized carbon atoms.

### Crystal data

**Table:**

[Pd(C_8_H_6_N_4_)_2_](BF_4_)_2_·C_2_H_3_N	*F*(000) = 2512
*M**_r_* = 637.41	*D*_x_ = 1.840 Mg m^−^^3^
Monoclinic, *C*2/*c*	Cu *K*α radiation, λ = 1.54178 Å
Hall symbol: -C 2yc	Cell parameters from 20829 reflections
*a* = 18.0686 (4) Å	θ = 3.6–68.8°
*b* = 18.1126 (4) Å	µ = 7.38 mm^−^^1^
*c* = 14.8351 (3) Å	*T* = 200 K
β = 108.613 (1)°	Block, yellow
*V* = 4601.13 (17) Å^3^	0.20 × 0.11 × 0.10 mm
*Z* = 8	

### Data collection

**Table:**

Bruker SMART 6000 diffractometer	4246 independent reflections
Radiation source: Rotating anode	4006 reflections with *I* > 2σ(*I*)
Montel 200 optics monochromator	*R*_int_ = 0.031
Detector resolution: 5.5 pixels mm^-1^	θ_max_ = 68.9°, θ_min_ = 3.6°
ω scans	*h* = −21→21
Absorption correction: multi-scan (*SADABS*; Sheldrick, 2004)	*k* = −20→21
*T*_min_ = 0.311, *T*_max_ = 0.478	*l* = −17→17
31076 measured reflections	

### Refinement

**Table:**

Refinement on *F*^2^	Secondary atom site location: difference Fourier map
Least-squares matrix: full	Hydrogen site location: inferred from neighbouring sites
*R*[*F*^2^ > 2σ(*F*^2^)] = 0.030	H-atom parameters constrained
*wR*(*F*^2^) = 0.081	*w* = 1/[σ^2^(*F*_o_^2^) + (0.0458*P*)^2^ + 11.6117*P*] where *P* = (*F*_o_^2^ + 2*F*_c_^2^)/3
*S* = 1.03	(Δ/σ)_max_ = 0.002
4246 reflections	Δρ_max_ = 1.07 e Å^−^^3^
347 parameters	Δρ_min_ = −0.75 e Å^−^^3^
0 restraints	Extinction correction: *SHELXL97* (Sheldrick, 2008), Fc^*^=kFc[1+0.001xFc^2^λ^3^/sin(2θ)]^-1/4^
Primary atom site location: structure-invariant direct methods	Extinction coefficient: 0.000355 (19)

### Special details

**Table:**

Experimental. X-ray crystallographic data for I were collected from a single-crystal sample, which was mounted on a loop fiber. Data were collected using a Bruker Platform diffractometer, equipped with a Bruker *SMART* 4 K Charged-Coupled Device (CCD) Area Detector using the program *APEX2* and a Nonius FR591 rotating anode equipped with Montel 200 optics. The crystal-to-detector distance was 5.0 cm, and the data collection was carried out in 512 *x* 512 pixel mode. The initial unit-cell parameters were determined by a least-squares fit of the angular setting of strong reflections, collected by a 10.0 degree scan in 33 frames over four different parts of the reciprocal space (132 frames total). One complete sphere of data was collected to better than 0.80 Å resolution.
Geometry. All e.s.d.'s (except the e.s.d. in the dihedral angle between two l. s. planes) were estimated using the full covariance matrix. The cell e.s.d.'s were taken into account individually in the estimation of e.s.d.'s in distances, angles, and torsion angles; correlations between e.s.d.'s in cell parameters were only used when they are defined by crystal symmetry. An approximate (isotropic) treatment of cell e.s.d.'s was used for estimating e.s.d.'s involving l. s. planes.
Refinement. Refinement of *F*^2^ against ALL reflections. The weighted *R*-factor *wR* and goodness-of-fit *S* are based on *F*^2^ and conventional *R*-factors *R* are based on *F*, with *F* set to zero for negative *F*^2^. The threshold expression of *F*^2^ > σ(*F*^2^) is used only for calculating *R*-factors(gt) *etc*. and is not relevant to the choice of reflections for refinement. *R*-factors based on *F*^2^ are statistically about twice as large as those based on *F*, and *R*-factors based on ALL data will be even larger.

### Fractional atomic coordinates and isotropic or equivalent isotropic displacement parameters (Å^2^)

**Table:**

	*x*	*y*	*z*	*U*_iso_*/*U*_eq_	
Pd1	0.5000	0.336135 (12)	0.2500	0.02227 (10)	
N1	0.44850 (12)	0.40551 (11)	0.13874 (15)	0.0244 (4)	
C1	0.46387 (15)	0.47710 (14)	0.13047 (19)	0.0294 (5)	
H1	0.5006	0.5018	0.1821	0.035*	
C2	0.42721 (17)	0.51534 (15)	0.0482 (2)	0.0359 (6)	
H2	0.4374	0.5662	0.0422	0.043*	
C3	0.37529 (18)	0.47724 (17)	−0.0249 (2)	0.0408 (7)	
H3	0.3476	0.5032	−0.0812	0.049*	
N3	0.36210 (15)	0.40471 (13)	−0.01994 (16)	0.0369 (5)	
C4	0.39986 (14)	0.37213 (14)	0.06120 (17)	0.0277 (5)	
C5	0.38836 (14)	0.29190 (14)	0.07200 (17)	0.0269 (5)	
N5	0.42993 (12)	0.26406 (11)	0.15719 (15)	0.0271 (4)	
C6	0.41328 (16)	0.19506 (14)	0.1769 (2)	0.0364 (6)	
H6	0.4384	0.1749	0.2381	0.044*	
C7	0.35997 (17)	0.15308 (15)	0.1090 (2)	0.0399 (7)	
H7	0.3477	0.1041	0.1223	0.048*	
C8	0.32526 (15)	0.18455 (16)	0.0217 (2)	0.0367 (6)	
H8	0.2909	0.1555	−0.0272	0.044*	
N8	0.33807 (13)	0.25488 (13)	0.00285 (16)	0.0340 (5)	
Pd2	0.0000	0.339164 (12)	0.2500	0.02379 (10)	
N9	0.06638 (12)	0.25370 (11)	0.23195 (15)	0.0278 (4)	
C9	0.13999 (16)	0.25769 (15)	0.2297 (2)	0.0349 (6)	
H9	0.1621	0.3045	0.2248	0.042*	
C10	0.18357 (18)	0.19496 (17)	0.2344 (2)	0.0429 (7)	
H10	0.2356	0.1974	0.2327	0.051*	
C11	0.14925 (18)	0.12859 (16)	0.2417 (2)	0.0433 (7)	
H11	0.1779	0.0844	0.2428	0.052*	
N11	0.07683 (15)	0.12380 (12)	0.24733 (18)	0.0382 (5)	
C12	0.03914 (15)	0.18649 (14)	0.24370 (18)	0.0287 (5)	
N12	0.04299 (12)	0.42452 (11)	0.19429 (15)	0.0257 (4)	
C13	0.07712 (16)	0.42029 (14)	0.12669 (19)	0.0314 (6)	
H13	0.0896	0.3733	0.1070	0.038*	
C14	0.09431 (17)	0.48312 (16)	0.0854 (2)	0.0364 (6)	
H14	0.1184	0.4806	0.0372	0.044*	
C15	0.07533 (17)	0.54960 (16)	0.1164 (2)	0.0393 (6)	
H15	0.0890	0.5937	0.0909	0.047*	
N15	0.03809 (14)	0.55468 (12)	0.18145 (17)	0.0349 (5)	
C16	0.02226 (14)	0.49198 (13)	0.21627 (18)	0.0274 (5)	
B1	0.2913 (2)	0.4231 (2)	0.2178 (3)	0.0447 (9)	
F1	0.27658 (12)	0.46150 (13)	0.13349 (14)	0.0621 (5)	
F2	0.36425 (10)	0.43957 (11)	0.27788 (13)	0.0496 (5)	
F3	0.28788 (19)	0.34813 (14)	0.19523 (19)	0.0962 (10)	
F4	0.23745 (14)	0.4387 (3)	0.26137 (17)	0.1125 (13)	
B2	0.0691 (2)	0.24786 (18)	0.5158 (2)	0.0373 (7)	
F5	0.09817 (13)	0.24491 (16)	0.44108 (15)	0.0749 (7)	
F6	0.00661 (12)	0.29637 (11)	0.49624 (17)	0.0644 (6)	
F7	0.04445 (17)	0.17898 (12)	0.5299 (2)	0.0885 (9)	
F8	0.12677 (16)	0.27183 (14)	0.59487 (17)	0.0869 (8)	
N16	0.35521 (18)	0.04534 (18)	0.5904 (2)	0.0563 (8)	
C17	0.3079 (2)	0.08800 (18)	0.5644 (2)	0.0446 (7)	
C18	0.2469 (2)	0.1432 (2)	0.5312 (4)	0.0771 (13)	
H18A	0.2098	0.1274	0.4703	0.116*	
H18B	0.2703	0.1905	0.5229	0.116*	
H18C	0.2195	0.1489	0.5781	0.116*	

### Atomic displacement parameters (Å^2^)

**Table:**

	*U*^11^	*U*^22^	*U*^33^	*U*^12^	*U*^13^	*U*^23^
Pd1	0.02316 (15)	0.01678 (15)	0.02304 (15)	0.000	0.00197 (10)	0.000
N1	0.0241 (10)	0.0227 (10)	0.0262 (10)	0.0030 (8)	0.0075 (8)	0.0017 (8)
C1	0.0293 (12)	0.0249 (13)	0.0352 (14)	0.0010 (10)	0.0118 (11)	0.0013 (10)
C2	0.0417 (15)	0.0280 (14)	0.0422 (15)	0.0048 (11)	0.0193 (13)	0.0103 (11)
C3	0.0503 (17)	0.0391 (16)	0.0316 (14)	0.0103 (13)	0.0112 (12)	0.0129 (12)
N3	0.0430 (13)	0.0380 (13)	0.0259 (11)	0.0062 (10)	0.0055 (10)	0.0034 (9)
C4	0.0266 (12)	0.0297 (13)	0.0269 (12)	0.0028 (10)	0.0086 (10)	−0.0010 (10)
C5	0.0244 (12)	0.0282 (13)	0.0277 (12)	0.0023 (10)	0.0076 (10)	−0.0030 (10)
N5	0.0262 (10)	0.0215 (10)	0.0294 (10)	0.0004 (8)	0.0030 (8)	−0.0033 (8)
C6	0.0362 (14)	0.0235 (13)	0.0415 (15)	−0.0010 (11)	0.0014 (12)	0.0010 (11)
C7	0.0330 (15)	0.0244 (13)	0.0557 (18)	−0.0030 (11)	0.0050 (13)	−0.0044 (12)
C8	0.0276 (13)	0.0324 (14)	0.0448 (16)	−0.0021 (11)	0.0041 (11)	−0.0160 (12)
N8	0.0284 (11)	0.0375 (13)	0.0320 (11)	0.0020 (9)	0.0039 (9)	−0.0083 (10)
Pd2	0.02831 (16)	0.01530 (15)	0.03000 (16)	0.000	0.01245 (11)	0.000
N9	0.0341 (11)	0.0218 (10)	0.0297 (10)	0.0020 (9)	0.0133 (9)	0.0000 (8)
C9	0.0360 (14)	0.0297 (14)	0.0423 (15)	0.0015 (11)	0.0169 (12)	0.0030 (11)
C10	0.0393 (16)	0.0415 (17)	0.0533 (18)	0.0082 (13)	0.0224 (14)	0.0001 (14)
C11	0.0466 (17)	0.0298 (15)	0.0564 (18)	0.0111 (12)	0.0204 (14)	−0.0024 (13)
N11	0.0448 (13)	0.0221 (11)	0.0487 (14)	0.0042 (10)	0.0161 (11)	−0.0022 (10)
C12	0.0351 (14)	0.0219 (12)	0.0296 (13)	−0.0001 (10)	0.0109 (10)	−0.0011 (10)
N12	0.0276 (11)	0.0205 (10)	0.0288 (10)	−0.0008 (8)	0.0084 (9)	0.0008 (8)
C13	0.0348 (14)	0.0276 (13)	0.0332 (14)	0.0006 (10)	0.0129 (11)	0.0015 (10)
C14	0.0379 (14)	0.0366 (15)	0.0375 (14)	−0.0008 (12)	0.0161 (12)	0.0067 (12)
C15	0.0422 (15)	0.0306 (14)	0.0464 (16)	−0.0036 (12)	0.0161 (13)	0.0106 (12)
N15	0.0413 (13)	0.0222 (11)	0.0414 (13)	−0.0026 (9)	0.0136 (10)	0.0031 (9)
C16	0.0291 (12)	0.0197 (12)	0.0303 (12)	−0.0006 (10)	0.0049 (10)	−0.0003 (10)
B1	0.0313 (17)	0.070 (2)	0.0338 (17)	−0.0178 (16)	0.0122 (14)	−0.0100 (16)
F1	0.0564 (12)	0.0816 (15)	0.0497 (11)	0.0054 (11)	0.0191 (9)	0.0139 (10)
F2	0.0344 (9)	0.0654 (12)	0.0463 (10)	−0.0109 (8)	0.0090 (8)	−0.0200 (9)
F3	0.128 (2)	0.0692 (16)	0.0691 (16)	−0.0514 (16)	−0.0002 (15)	−0.0088 (12)
F4	0.0420 (12)	0.251 (4)	0.0502 (13)	0.0151 (18)	0.0228 (11)	−0.0055 (18)
B2	0.0413 (17)	0.0302 (16)	0.0408 (17)	0.0011 (13)	0.0135 (14)	0.0002 (13)
F5	0.0584 (13)	0.119 (2)	0.0545 (12)	0.0111 (13)	0.0274 (10)	0.0160 (13)
F6	0.0525 (12)	0.0496 (12)	0.0871 (15)	0.0149 (9)	0.0167 (11)	0.0021 (11)
F7	0.105 (2)	0.0334 (10)	0.157 (3)	−0.0061 (12)	0.084 (2)	−0.0008 (14)
F8	0.0868 (17)	0.0793 (17)	0.0655 (14)	0.0202 (13)	−0.0165 (12)	−0.0219 (12)
N16	0.0577 (18)	0.070 (2)	0.0405 (15)	0.0225 (16)	0.0146 (13)	−0.0056 (14)
C17	0.0408 (17)	0.052 (2)	0.0403 (17)	0.0031 (14)	0.0113 (14)	−0.0006 (13)
C18	0.047 (2)	0.060 (2)	0.117 (4)	0.0100 (19)	0.017 (2)	0.030 (3)

### Geometric parameters (Å, º)

**Table:**

Pd1—N5^i^	2.022 (2)	C9—H9	0.9500
Pd1—N5	2.022 (2)	C10—C11	1.372 (5)
Pd1—N1	2.047 (2)	C10—H10	0.9500
Pd1—N1^i^	2.047 (2)	C11—N11	1.340 (4)
N1—C1	1.340 (3)	C11—H11	0.9500
N1—C4	1.348 (3)	N11—C12	1.316 (3)
C1—C2	1.375 (4)	C12—C12^ii^	1.485 (5)
C1—H1	0.9500	N12—C13	1.337 (3)
C2—C3	1.372 (4)	N12—C16	1.348 (3)
C2—H2	0.9500	C13—C14	1.374 (4)
C3—N3	1.341 (4)	C13—H13	0.9500
C3—H3	0.9500	C14—C15	1.371 (4)
N3—C4	1.318 (3)	C14—H14	0.9500
C4—C5	1.484 (4)	C15—N15	1.345 (4)
C5—N8	1.316 (3)	C15—H15	0.9500
C5—N5	1.345 (3)	N15—C16	1.316 (3)
N5—C6	1.339 (3)	C16—C16^ii^	1.471 (5)
C6—C7	1.378 (4)	B1—F4	1.357 (4)
C6—H6	0.9500	B1—F2	1.368 (4)
C7—C8	1.369 (4)	B1—F1	1.381 (4)
C7—H7	0.9500	B1—F3	1.396 (5)
C8—N8	1.340 (4)	B2—F7	1.363 (4)
C8—H8	0.9500	B2—F8	1.368 (4)
Pd2—N12	2.021 (2)	B2—F5	1.371 (4)
Pd2—N12^ii^	2.021 (2)	B2—F6	1.386 (4)
Pd2—N9	2.028 (2)	N16—C17	1.125 (4)
Pd2—N9^ii^	2.028 (2)	C17—C18	1.452 (5)
N9—C9	1.343 (3)	C18—H18a	0.9800
N9—C12	1.345 (3)	C18—H18b	0.9800
C9—C10	1.372 (4)	C18—H18c	0.9800
			
N5^i^—PD1—N5	99.59 (11)	N9—C9—H9	119.6
N5^i^—PD1—N1	165.94 (8)	C10—C9—H9	119.6
N5—PD1—N1	79.78 (8)	C9—C10—C11	117.6 (3)
N5^i^—PD1—N1^i^	79.78 (8)	C9—C10—H10	121.2
N5—PD1—N1^i^	165.94 (8)	C11—C10—H10	121.2
N1—PD1—N1^i^	104.24 (11)	N11—C11—C10	122.4 (3)
C1—N1—C4	117.0 (2)	N11—C11—H11	118.8
C1—N1—PD1	127.87 (17)	C10—C11—H11	118.8
C4—N1—PD1	114.69 (16)	C12—N11—C11	116.4 (2)
N1—C1—C2	120.9 (2)	N11—C12—N9	125.4 (2)
N1—C1—H1	119.6	N11—C12—C12^ii^	119.74 (16)
C2—C1—H1	119.6	N9—C12—C12^ii^	114.78 (14)
C3—C2—C1	117.5 (3)	C13—N12—C16	117.6 (2)
C3—C2—H2	121.2	C13—N12—PD2	126.33 (17)
C1—C2—H2	121.2	C16—N12—PD2	114.94 (17)
N3—C3—C2	122.5 (3)	N12—C13—C14	120.7 (2)
N3—C3—H3	118.7	N12—C13—H13	119.6
C2—C3—H3	118.7	C14—C13—H13	119.6
C4—N3—C3	116.1 (2)	C15—C14—C13	117.5 (3)
N3—C4—N1	125.8 (2)	C15—C14—H14	121.2
N3—C4—C5	119.2 (2)	C13—C14—H14	121.2
N1—C4—C5	115.0 (2)	N15—C15—C14	122.5 (2)
N8—C5—N5	125.5 (2)	N15—C15—H15	118.8
N8—C5—C4	120.0 (2)	C14—C15—H15	118.8
N5—C5—C4	114.4 (2)	C16—N15—C15	116.3 (2)
C6—N5—C5	117.2 (2)	N15—C16—N12	125.1 (2)
C6—N5—PD1	126.12 (18)	N15—C16—C16^ii^	120.10 (15)
C5—N5—PD1	115.98 (16)	N12—C16—C16^ii^	114.78 (14)
N5—C6—C7	120.6 (3)	F4—B1—F2	109.5 (3)
N5—C6—H6	119.7	F4—B1—F1	111.5 (3)
C7—C6—H6	119.7	F2—B1—F1	110.3 (3)
C8—C7—C6	117.6 (3)	F4—B1—F3	109.5 (3)
C8—C7—H7	121.2	F2—B1—F3	109.1 (3)
C6—C7—H7	121.2	F1—B1—F3	106.9 (3)
N8—C8—C7	122.4 (2)	F7—B2—F8	110.8 (3)
N8—C8—H8	118.8	F7—B2—F5	108.5 (3)
C7—C8—H8	118.8	F8—B2—F5	108.8 (3)
C5—N8—C8	116.3 (2)	F7—B2—F6	109.2 (3)
N12—PD2—N12^ii^	80.22 (12)	F8—B2—F6	109.0 (3)
N12—PD2—N9	102.08 (8)	F5—B2—F6	110.6 (3)
N12^ii^—PD2—N9	163.44 (9)	N16—C17—C18	179.7 (4)
N12—PD2—N9^ii^	163.44 (9)	C17—C18—H18A	109.5
N12^ii^—PD2—N9^ii^	102.08 (8)	C17—C18—H18B	109.5
N9—PD2—N9^ii^	80.47 (12)	H18A—C18—H18B	109.5
C9—N9—C12	117.3 (2)	C17—C18—H18C	109.5
C9—N9—PD2	126.46 (18)	H18A—C18—H18C	109.5
C12—N9—PD2	114.67 (17)	H18B—C18—H18C	109.5
N9—C9—C10	120.7 (3)		

Symmetry codes: (i) −*x*+1, *y*, −*z*+1/2; (ii) −*x*, *y*, −*z*+1/2.

### Hydrogen-bond geometry (Å, º)

**Table:**

*D*—H···*A*	*D*—H	H···*A*	*D*···*A*	*D*—H···*A*
C1—H1···N11^iii^	0.95	2.62	3.460 (4)	148
C6—H6···N15^iv^	0.95	2.45	3.237 (4)	140
C14—H14···N16^v^	0.95	2.40	3.228 (4)	146
C2—H2···F7^v^	0.95	2.38	3.282 (3)	159
C3—H3···F2^vi^	0.95	2.44	3.240 (3)	142
C8—H8···F3^vii^	0.95	2.45	3.270 (4)	145
C13—H13···F6^ii^	0.95	2.37	2.982 (3)	122
C15—H15···F8^vi^	0.95	2.52	3.408 (4)	155
C18—H18*B*···F5^viii^	0.98	2.55	3.373 (5)	142

Symmetry codes: (ii) −*x*, *y*, −*z*+1/2; (iii) *x*+1/2, *y*+1/2, *z*; (iv) −*x*+1/2, *y*−1/2, −*z*+1/2; (v) −*x*+1/2, *y*+1/2, −*z*+1/2; (vi) *x*, −*y*+1, *z*−1/2; (vii) −*x*+1/2, −*y*+1/2, −*z*; (viii) −*x*+1/2, −*y*+1/2, −*z*+1.
